# Wie wirken Generikaquoten? Eine Analyse am Beispiel der HIV-Infektion

**DOI:** 10.1007/s00103-021-03312-1

**Published:** 2021-04-14

**Authors:** Robin Rüsenberg, Axel Baumgarten, Stefan Mauss, Gabriele Gradl, Martin Schulz, Barbara Bartmeyer, Christian Kollan, Daniel Schmidt

**Affiliations:** 1 Deutsche Arbeitsgemeinschaft niedergelassener Ärzte in der Versorgung HIV-Infizierter e. V. (dagnä), Berlin, Deutschland; 2 Zentrum für Infektiologie Berlin Prenzlauer Berg (ZIBP), Berlin, Deutschland; 3 Zentrum für HIV und Hepatogastroenterologie, Düsseldorf, Deutschland; 4 Deutsches Arzneiprüfungsinstitut e. V. (DAPI), Berlin, Deutschland; 5 Institut für Pharmazie, Freie Universität Berlin, Berlin, Deutschland; 6 Geschäftsbereich Arzneimittel, ABDA – Bundesvereinigung Deutscher Apothekerverbände e. V., Berlin, Deutschland; 7 Abteilung für Infektionsepidemiologie, Robert Koch-Institut, Seestraße 10, 13353 Berlin, Deutschland

**Keywords:** Arzneimittelversorgung, Versorgungssteuerung, Ambulante Versorgung, Sekundärdaten, Gesetzliche Krankenversicherung (GKV), Medical supply, Managed primary care, Ambulatory healthcare, Secondary data, Statutory health insurance

## Abstract

**Hintergrund:**

Die Steuerungsinstrumente der Arzneimittelversorgung in der gesetzlichen Krankenversicherung (GKV) sind schon länger Bestandteil der gesundheitspolitischen Reformdebatte. Über die Jahre hat sich eine Gemengelage sehr verschiedener Werkzeuge herausgebildet, die zumeist auf eine Kontrolle der Arzneimittelausgaben zielen. Die Instrumente der regionalen Verordnungssteuerung fokussieren vor allem auf eine Verhaltenssteuerung des verordnenden Arztes. Zu erwähnen ist nicht zuletzt der verstärkte Einsatz von indikationsbezogenen Quoten, vorrangig Leitsubstanzen und/oder Generika/Biosimilars. Diese gibt es mittlerweile auch im Bereich des Humanen Immundefizienz-Virus (HIV), etwa die seit 2020 in Bayern und Berlin eingeführten Generikaquoten für HIV-Therapeutika.

**Zielstellung:**

Ziel des vorliegenden Beitrages ist es, auf Grundlage von GKV-Apothekenabrechnungsdaten das Potenzial sowie Grenzen von Generikaquotenlösungen in der HIV-Versorgung zu analysieren und Handlungsempfehlungen zu skizzieren.

**Ergebnisse:**

Es zeigte sich, dass das Quotenpotenzial für Generika im patentfreien Bereich in der HIV-Versorgung bereits weitgehend ausgeschöpft wird. Dieser Umstand ist vor allem darauf zurückzuführen, dass die HIV-Verordner den Austausch durch Verzicht auf Aut-idem-Kreuze auf dem Kassenrezept unterstützen.

**Diskussion:**

Das steuerungspolitische Optimum ist fast erreicht. Dies ist auf das geeignete Instrumentarium zurückzuführen, das aus dem Rahmenvertrag zur Arzneimittelversorgung und einer leitliniengerechten Wirkstoffverordnung durch den Arzt besteht – in Verbindung mit dem AMNOG(Arzneimittelmarktneuordnungsgesetz)-Verfahren und Festbeträgen. Leitlinienkonformität und (existierende) Eintablettenregime müssen beibehalten werden, damit die gute Versorgungsqualität gewährleistet bleibt.

## Abstract (en)

**Background:**

Instruments controlling statutory healthcare medical supply have long been a topic of debate in health policy reform discussions. Over the years, a variety of tools have been developed, most of which are aimed at controlling drug expenditure. The instruments controlling regional prescriptions primarily focus on controlling behavioural patterns of the prescribing physicians. Important to note is the increased use of indication-directed quotas, primarily of drug leads and/or generics/biosimilars. These are now also available in the area of the human immunodeficiency virus (HIV), such as the generic quotas for HIV medications introduced in Bavaria and Berlin in 2020.

**Objective:**

The aim of this article is to analyse the benefits and limitations of generic quota solutions in HIV care using statutory health insurance drug prescription data and to outline recommendations for action.

**Results:**

It was observed that the quota potential for generics in the area of patent-free drugs in HIV care has already been largely exhausted. This can be explained by HIV prescribers supporting product exchange on the prescription.

**Discussion:**

The best-case scenario in terms of regulation has almost been reached. This is due to a suitable set of instruments, including the framework agreement for medical supply as well as prescribing according to guidelines – in conjunction with the Pharmaceuticals Market Reorganisation Act (AMNOG) and reference prices for drugs. Conforming with guidelines and (existing) single-tablet regimens play an integral role in maintaining good quality of care.

## Einleitung

Die Steuerungsinstrumente der Arzneimittelversorgung in der gesetzlichen Krankenversicherung (GKV) sind schon länger Bestandteil der gesundheitspolitischen Reformdebatte. Über die Jahre hat sich eine Gemengelage sehr verschiedener Werkzeuge herausgebildet, die zumeist auf eine Kontrolle der Arzneimittelausgaben zielen. Dabei kann es sich um gesetzliche oder vertragliche, kollektive oder selektivvertragliche, bei verschiedenen Akteuren ansetzende oder weitere Maßnahmen handeln, darunter etwa die Anteile von Aut-idem-Kreuzen auf den Kassenrezepten, Erstattungsbeträge, Festbeträge und Rabattverträge, die sich wiederum nach den inhaltlichen Ansatzpunkten – Preis, Menge, Struktur – unterscheiden.

Das Steuerungsinstrumentarium wirkt auch in der Versorgung von Menschen mit dem Humanen Immundefizienz-Virus (HIV), deren Therapie im Wesentlichen in Form der antiretroviralen Therapie (ART) medikamentös erfolgt: Die Preisbildung reguliert vornehmlich das sog. AMNOG(Arzneimittelmarktneuordnungsgesetz)-Verfahren, das durch eine frühe Nutzenbewertung und anschließende Erstattungsbetragsverhandlungen gekennzeichnet ist. Von 2011 bis Ende 2020 gab es 21 Nutzenbewertungen im Bereich HIV, deren inhaltliche Ergebnisse auch direkt das Verordnungsverhalten der Schwerpunktärzte beeinflussen [1]. Ebenfalls den Preis nehmen die bereits seit 1989 existierenden Festbeträge in den Fokus. Im Jahr 2016 hat der Gemeinsame Bundesauschuss (G-BA) mit der Bildung von Festbeträgen für die HIV-Indikation begonnen (Zidovudin + Lamivudin, Efavirenz) – noch ohne größere Auswirkungen auf die Versorgungsrealität. Abzuwarten bleibt, wie die neue Festbetragsgruppe „Kombinationen zweier Nukleos(t)id-Analoga“ (Tenofovir-Disoproxil/Emtricitabin TDF/FTC, Abacavir/Lamivudin ABC/3TC und Tenofovir-Alafenamid/Emtricitabin TAF/FTC) sowie des Proteaseinhibitors Darunavir (DRV) seit Oktober 2020 wirkt [2].

Bereits umfangreichere Erfahrungen gibt es in der HIV-Schwerpunktversorgung mit (ebenfalls preisregulierenden) Rabattverträgen, die in der Regel bei der Abgabe in der Apotheke ansetzen. Mittlerweile informieren zudem Krankenkassen verstärkt die HIV-Schwerpunktärzte über die von ihnen abgeschlossenen Rabattverträge. Zusätzlich existieren vielfältige Instrumente der regionalen Verordnungssteuerung, die den Fokus auf Struktur und/oder Menge und damit vor allem auf Verhaltenssteuerung bei dem verordnenden Arzt setzen. Zu erwähnen sind vor allem die seit 2020 über die jeweilige regionale Kassenärztliche Vereinigung (KV) eingeführten Generikaquoten für HIV-Therapeutika in Bayern und Berlin. Auf der Ebene der regionalen Arzneimittelvereinbarungen zeigt sich damit immer deutlicher ein Paradigmenwechsel: einerseits eine Abkehr von statistischen Auffälligkeitsprüfungen, etwa Richtgrößen oder anhand von Durchschnittswerten, andererseits eine Hinwendung zu indikationsbezogenen Quoten, vorrangig Leitsubstanzen und/oder Generika/Biosimilars.

Eine entscheidende Rolle bei der Arzneimittelregulierung durch Quoten nimmt, wenig überraschend, das Verordnungsverhalten der Vertragsärzte ein. Ziel des vorliegenden Beitrages ist es, auf Grundlage von GKV-Apothekenabrechnungsdaten das Potenzial sowie Grenzen von Generikaquotenlösungen in der Versorgung von Menschen mit HIV zu analysieren und Handlungsempfehlungen zu skizzieren. Dabei sollen ebenfalls der (nachfragesteuernde) Status quo in der HIV-Arzneimittelversorgung sowie das Instrument der Rabattverträge beschrieben werden.

## HIV-Arzneimittelversorgung und verordnungssteuernde Maßnahmen

Die ambulante HIV-Schwerpunktversorgung in Deutschland ist überaus erfolgreich – der medizinische Goldstandard, also die effektive Absenkung der Viruslast im Blut unter die Nachweisbarkeitsgrenze, wird bei 96 % der Betroffenen in Therapie erreicht [3]. Die Versorgung ist aber auch kostenintensiv. Im Schnitt hatte die GKV im Jahr 2018 Leistungsausgaben von ca. 3100 €/Jahr pro Versicherten [4]; die Versorgung von Patienten, die mit HIV infiziert sind, ist finanziell aufwendiger. Für HIV-Patienten sind es – bezogen auf den Zeitraum 2014–2017 – im Schnitt 17.300 €/Jahr, woran die HIV-Therapeutika einen Anteil von ca. 85 % haben [5]. Seit 2018 sind die Kosten infolge von Preisanpassungen gesunken.

Die ART-Standardtherapie setzt als Kombinationstherapie i. d. R. auf 3 antiretrovirale Substanzen aus mindestens 2 Wirkstoffklassen der Nukleos(t)ide-Reverse-Transkriptase-Inhibitoren (NRTI) sowie entweder Non-Nukleos(t)ide-Reverse-Transkriptase-Inhibitoren (NNRTI), Proteaseinhibitoren (PI) oder Integrase-Strang-Transfer-Inhibitoren (INSTI; [6]). Mittlerweile werden diese oftmals zusammen in einer Tablette verabreicht (Eintablettenregime, Single-Tablet-Regimen – STR). Dies zeigt sich auch in gesunkenen Absatzmengen bezogen auf die Tagesdosen [7]. Seit Kurzem wurden zusätzlich 2 duale STR-Regime im Markt platziert, die mittlerweile einen nennenswerten Umsatz haben. Anfang 2020 wurden 40 Präparate zur Behandlung der HIV-Infektion in Deutschland verordnet, von denen wiederum knapp ein Drittel generisch verfügbar war. Es gilt allerdings zu bedenken, dass der Einsatz der meisten ART-Generika – die es grundsätzlich seit 2013 gibt – nicht mehr durch die Leitlinien empfohlen wird [8]. Vor allem seit dem Fall des Patents von TDF bzw. TDF/FTC im Juli 2017 ist eine deutliche Marktdynamik zu spüren, die sich in gesunkenen Tagestherapiekosten niederschlägt. TDF ist Bestandteil von Erstlinientherapeutika und der Präexpositionsprophylaxe der HIV-Infektion (PrEP). Anfang 2019 lag die ART-Generikaquote bei knapp über 20 % [1, 9, 10].

Unmittelbar auf das generische Segment zielen Rabattverträge nach § 130a Abs. 8 Fünftes Buch Sozialgesetzbuch (SGB V). Diese erfreuen sich bei Krankenkassen großer Beliebtheit, da sie direkte Einsparungen genieren (2019: 4,9 Mrd. €; [11]); sie werden auch in der privaten Krankenversicherung (PKV) genutzt. Als angebotsorientiertes Steuerungsinstrument setzen sie beim Faktor Preis an und werden als Teil des Vertragswettbewerbs direkt zwischen Krankenkasse und pharmazeutischem Unternehmen vereinbart. Den entscheidenden Schub erhielt das Instrument Rabattverträge durch die seit 2007 geltende Verpflichtung der Apotheken, die kassenspezifischen Rabattverträge vorrangig zu bedienen, sofern der Verordnende den Austausch (durch Verzicht auf das Setzen eines Aut-idem-Kreuzes in der Verordnung) zugelassen hat [12]. Auch im Segment der HIV-Therapeutika existiert eine Vielzahl an Rabattverträgen. Detaillierte Aussagen sind aufgrund der Vertraulichkeit der Vereinbarungen allerdings schwer zu treffen. Über das Arztinformationssystem wird der Verordnende aber über das Vorhandensein eines Rabattvertrages informiert. Auch die einzelnen Krankenkassen informieren darüber. Das Vorhandensein von Rabattverträgen hat schließlich bei fast 66 % der HIV-Schwerpunktpraxen Einfluss auf das Verordnungsverhalten [1]. Hilfreich ist, dass Krankenkassen für rabattierte Arzneimittel i. d. R. eine „Wirtschaftlichkeitsgarantie“ geben, d. h., die Verordnung gilt immer als wirtschaftlich, wodurch der verordnende Arzt vor Regressforderungen durch die Krankenkasse geschützt ist [13].

Auch in den regionalen Arzneimittelvereinbarungen nach § 84 SGB V finden Rabattverträge oft Berücksichtigung. Diese sind das zentrale Instrument der Nachfragesteuerung. Zwar existieren ebenso Informationsschreiben von KV und/oder Krankenkassen, im Mittelpunkt stehen jedoch die regional zwischen KV und den Landesverbänden der Krankenkassen und den Ersatzkassen vertraglich festgelegten Regelungen. Diese werden durch regelmäßige Wirtschaftlichkeitsprüfungen nach § 106 SGB V umgesetzt. Bis Ende 2016 war die sog. Richtgrößenprüfung (Auffälligkeitsprüfung) als Regelprüfmethode vorgesehen, also ein arztgruppenspezifisches, statistisches Prüfverfahren mit normativem Charakter. Die HIV-Therapie war in allen KV-Regionen dabei als Praxisbesonderheit definiert, um dem Umstand Rechnung zu tragen, dass die ART in den Schwerpunktpraxen deutlich höhere Kosten verursachte als in den Praxen derselben Fachgruppe ohne HIV-Schwerpunkt (Stichwort „atypische Hausarztpraxen“). Ergänzend waren Höchst- und Mindestquoten, mal als Orientierung, mal mit Auswirkung auf die Richtgrößenprüfung, möglich. Etwa 2016 in der KV-Region Nordrhein, wo man sich den „Abbau von Fehl‑, Über- und Unterversorgung insbesondere im Bereich … der Arzneimittel zur Therapie von HIV-Infektionen“ vornahm; allerdings ohne konkrete Umsetzungsschritte [14].

Zu Januar 2017 wurden die Rahmenbedingungen durch das GKV-Versorgungsstärkungsgesetz (GKV-VSG) regionalisiert. Seitdem ist die Richtgrößenprüfung nicht mehr obligatorisch – vielmehr können die regionalen Vertragspartner eigene Prüfungsarten und -methoden umsetzen, was im Ergebnis zu einem bunten Strauß an verordnungssteuernden Maßnahmen geführt hat. Insgesamt zeigt sich jedoch ein Trend zu indikationsbezogenen Quoten, vorrangig Leitsubstanzen und/oder Generika/Biosimilars (Zielquoten werden dabei selbst in denjenigen KV-Regionen, die noch auf Auffälligkeitsprüfungen setzen, als Ergänzung eingesetzt). Auch in der Versorgung von Menschen mit HIV findet dies langsam Niederschlag: Ende 2020 war in 15 von 17 KV-Regionen entweder – dort, wo nach statistischen Verfahren geprüft wird – HIV als Praxisbesonderheit anerkannt. Oder aber die Regelungen in den „Quoten“-Regionen sahen keine Vorgaben für die ART vor.

Eine Ausnahme bildeten Bayern und Berlin: Die bayerische Wirkstoffvereinbarung sieht eine facharztgruppenspezifische Verordnungsquote für „HIV-Therapeutika“ vor (Generikaquote am ART-Gesamtmarkt inklusive TDF zur Therapie der chronischen Hepatitis B [HBV] und TDF/FTC als PrEP). Jede Vergleichsgruppe erhält pro Wirkstoffgruppe eine Zielquote, z. B. 37 % bei Hausärzten, 49,9 % bei Internisten ohne Schwerpunkt, 89,2 % bei fachärztlichen Internisten mit Spezialisierung Gastroenterologie sowie etwa 43 % bei den meisten weiteren Arztgruppen, wobei Rabattverträge berücksichtigt werden [15]. Insgesamt werden arztgruppenspezifische Zielquoten definiert, deren Erfüllung weitere arztindividuelle Prüfungen obsolet macht.

In Berlin hingegen setzen die regionalen Vertragspartner zwar weiterhin auf ein statistisches Verfahren (Durchschnittswerteprüfung), allerdings fallen die meisten Praxisbesonderheiten inkl. HIV weg. Dafür wird für HIV-Schwerpunktärzte eine Generikaquote von 17,86 % am ART-Gesamtmarkt definiert [16], deren arztindividuelles Erreichen dazu führt, dass die HIV-Therapeutika komplett aus der Durchschnittswerteprüfung herausgenommen werden – und damit quasi wieder als Praxisbesonderheit gelten. Rabattverträge werden bei der Quotenerfüllung in Berlin nicht, dafür aber in Bayern berücksichtigt.

Bayern (reines Quotenmodell) und Berlin (Auffälligkeitsprüfung, aber Berücksichtigung von Quoten) stehen damit auch stellvertretend für die beiden Trends in der regionalen Verordnungssteuerung. Ein dritter Weg sind Selektivverträge, um auf das Verordnungsverhalten des Arztes Einfluss zu nehmen. So ist im Oktober 2020 ein Selektivvertrag nach § 140a SGB V zur Optimierung der Versorgung von Menschen mit HIV in der KV-Region Nordrhein in Kraft getreten, der u. a. einen Arzneimittelcheck vorsieht [17].

## Methodik

Die im vorliegenden Beitrag genutzten Sekundärdatenquellen sind Verordnungsdaten aus Apothekenabrechnungszentren von Personen mit gesetzlicher Krankenversicherung. Dabei handelte es sich einmal um Daten des Dienstleisters INSIGHT Health GmbH & Co. KG mit einer vom Anbieter angegebenen Abdeckung der öffentlichen Apotheken in allen Regionen Deutschlands von > 99 % sowie um Daten des Deutschen Arzneiprüfungsinstituts e. V. (DAPI) mit einer Abdeckung von mehr als 80 % (bis Juni 2019) bzw. über 95 % (ab Juli 2019), welche auf 100 % hochgerechnet wurden. Die GKV repräsentiert rund 87 % der deutschen Bevölkerung. Die ausgewerteten Daten enthielten abgerechnete Verordnungen der in Apotheken eingelösten Rezepte gegen die Infektionskrankheiten HIV und HBV. Bei der Behandlungsindikation ist zu beachten, dass die Medikamente – TDF und die Kombination TDF/FTC – für 2 Einsatzgebiete die Marktzulassung besitzen: Ersteres für die Behandlung von HIV und HBV, Letzteres zur HIV-Behandlung als auch zur PrEP. Für welche Indikation verordnet wurde, lässt sich anhand der Abrechnungsdaten nicht ermitteln. In den weiteren Betrachtungen wird von der vereinfachenden, aber nicht ganz korrekten Annahme ausgegangen, dass TDF als Einzelsubstanz vollständig der HBV-Behandlung zuzuordnen ist.

Die Apothekenabrechnungsdaten wurden ausgewertet im Hinblick auf die Anteile von patentgeschützten Präparaten, Altoriginalen (nicht mehr patentgeschützte Erstanbieterprodukte), hierzu verfügbaren Generika. Zudem wurden sie auf bestimmte Steuerungsinstrumente wie den Rahmenvertrag über die Arzneimittelversorgung nach § 129 Abs. 2 SGB V (Abgaben von Rabattarzneimitteln, Abgaben mit gesetztem Aut-idem-Kreuz) untersucht. Ziel ist es, auf Basis der Fragestellungen des Beitrages das Verordnungsverhalten der jüngeren Zeit abzubilden. Es wurde zunächst ein Blick auf den Status quo für den Zeitraum 2013 bis 2020 vorgenommen. Die Anteile von Generika am Gesamtmarkt auf Basis der definierten Tagesdosen (Defined Daily Dose [DDD]) für HIV und HBV wurden in Berlin, Bayern und den übrigen KV-Regionen ermittelt.^4^ Darüber hinaus wurden Quotenszenarien unter Berücksichtigung der aktuellen Deutsch-Österreichischen Leitlinien zur antiretroviralen Therapie der HIV-Infektion [8] entwickelt, um den Einfluss einer Generikaquote auf die Therapiewahl zu prüfen.

## Ergebnisse

In Tab. 1 ist der generische Anteil an den jährlichen Gesamt-DDDs für die KV Bayern, Berlin und die weiteren 15 KV-Regionen nach Krankheitsindikation dargestellt. Hierbei ist zu bedenken, dass in der bayerischen Wirkstoffvereinbarung zwar von HIV-Therapeutika gesprochen wird, aber für die Generikaziele in dieser Kategorie alle Medikamente sowohl für die Behandlung und Prophylaxe von HIV als auch der HBV zusammengefasst wurden (Spalte „Gesamt“).

**Table 1:** 

	HIV	HIV	HBV	HBV	Gesamt	Gesamt
	DDD in Mio.	Generisch (in %)	DDD in Mio.	Generisch (in %)	DDD in Mio.	Generisch (in %)
*KV Bayern*						
2013	4,60	0,5	0,85	5,2	5,46	1,2
2014	4,73	1,5	0,91	4,8	5,65	2,0
2015	4,61	2,3	0,93	5,1	5,54	2,8
2016	4,57	2,6	1,01	5,5	5,58	3,2
2017	4,58	4,6	1,09	28,4	5,67	9,2
2018	4,31	12,0	1,14	59,2	5,45	21,9
2019	4,5	25,4	1,24	81,4	5,74	37,4
2020	2,31	30,9	0,63	85,4	2,94	42,5
*KV Berlin*						
2013	7,64	0,5	0,63	4,5	8,27	0,8
2014	7,82	2,0	0,67	7,3	8,49	2,4
2015	7,36	2,4	0,66	7,6	8,02	2,8
2016	6,54	2,8	0,65	8,7	7,19	3,3
2017	6,53	5,7	0,66	30,3	7,19	8,0
2018	6,22	14,0	0,68	64,4	6,89	18,9
2019	5,47	28,0	0,70	86,2	6,17	34,7
2020	3,07	37,2	0,36	89,3	3,44	42,7
*KV andere*						
2013	22,63	0,7	5,06	3,1	27,69	1,1
2014	23,04	2,5	5,48	6,3	28,52	3,2
2015	23,05	3,2	5,69	8,6	28,74	4,2
2016	23,96	3,7	6,06	9,5	30,01	4,9
2017	23,84	6,4	6,38	29,0	30,22	11,1
2018	22,22	14,8	6,64	62,7	28,86	25,8
2019	20,7	25,4	7,00	83,0	27,7	39,9
2020	11,63	30,9	3,63	87,9	15,27	44,5
*Bundesweit*						
2013	34,87	0,6	6,55	3,5	41,42	1,1
2014	35,59	2,3	7,07	6,2	42,66	2,9
2015	35,03	2,9	7,28	8,0	42,31	3,8
2016	35,07	3,4	7,72	8,9	42,79	4,4
2017	34,95	6,0	8,13	29,0	43,08	10,4
2018	32,75	14,2	8,46	62,3	41,20	24,1
2019	30,68	25,8	8,94	83,0	39,62	38,7
2020	17,02	32,0	4,62	87,6	21,65	43,9

**Figure 1:**
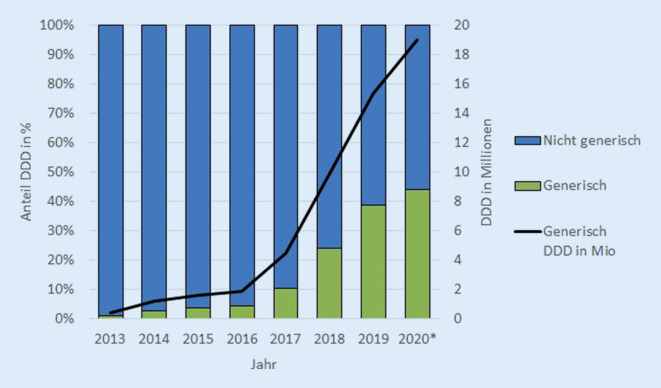

Es war ein deutlicher Anstieg der Generikaquote über die letzten Jahre zu verzeichnen (Abb. 1). Im Jahr 2020 lag die Generikaquote im HIV-Bereich in Bayern und dem restlichen Bundesgebiet bei 31 %, in Berlin bei 37 %. Trotz des sprunghaften Anstiegs von generischem TDF/FTC sowohl in der HIV-Therapie als auch durch die PrEP seit September 2019 liegt die erreichte Quote für den HIV-Bereich im Jahr 2020 bei unter 40 %. Aufgrund des starken Anteils des seit 2017 generischen TDF in der HBV-Therapie und der Tatsache, dass auch Entecavir generisch ist, wurden bei dieser Indikation nun Quoten von bis zu fast 90 % erreicht. Damit wurde für die Gesamtheit der HIV- und HBV-Therapeutika eine Quote von 44 % im Jahr 2020 erreicht (Tab. 1; Abb. 1). Bei einer vollständig generischen Gabe der HBV-Therapie steigt die generische Gesamtquote über alle 3 Indikationen noch um 3 %.

**Figure 2:**
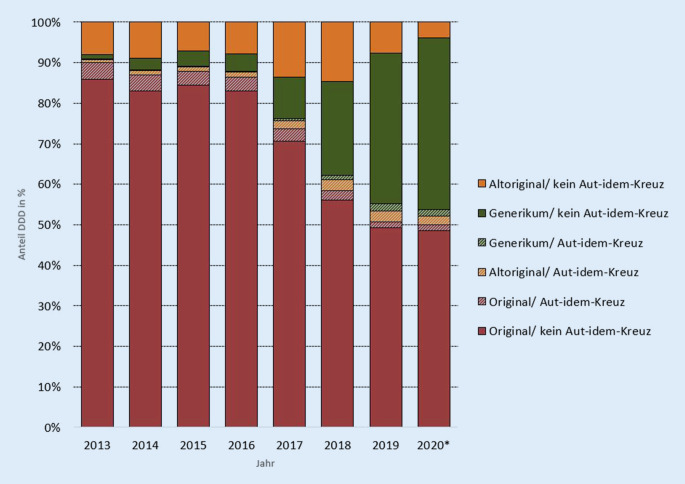

Welche aktiven Handlungsoptionen bieten sich nun aber unter den Vorgaben einer Arzneimittelvereinbarung, deren zentrale Steuerungselemente Generikaquoten und Rabattverträge sind? Wie erwähnt gilt für Apotheken die Verpflichtung, die kassenspezifischen Rabattverträge vorrangig zu bedienen, sofern der Verordnende den Austausch zulässt. Es wurden also die Fälle betrachtet, in denen dem Apotheker vom Verordnenden explizit untersagt wurde, Medikamente gegen ein wirkstoffgleiches Präparat zu tauschen (Abb. 2). Das im gegenteiligen Wortsinn sogenannte Aut-idem-Kreuz auf dem Rezept kann sich, je nach Wahl, sowohl im positiven als auch im negativen Sinne auf die Generikaquote auswirken.

Der Blick auf Apothekendaten zeigt, dass nur rund 5 % der generikafähigen Medikamente in den Jahren 2013–2020 als nicht austauschfähig markiert wurden (Abb. 2). Die verordnenden Ärzte unterstützen somit das Steuerungselement Rabattvertrag. Hierbei ist noch anzumerken, dass nicht nur Altoriginale per Aut-idem-Kreuz in den Verordnungen gehalten wurden, sondern im Gegenteil auch eine Fixierung auf bestimmte Generika durch Setzen des Aut-idem-Kreuzes stattfand.

Eine andere Möglichkeit der aktiven Einflussnahme ist die Wahl der Wirkstoffe bzw. Medikamente in der Therapie. Betrachten wir daher, in welchem Verhältnis Originale bzw. Altoriginale und Generika in der HIV-Therapie stehen unter Berücksichtigung der in der aktuellen Deutsch-Österreichischen Leitlinie zur antiretroviralen Therapie der HIV-Infektion empfohlenen Substanzkombinationen [8]. Als generikafähige Stoffe bieten sich hier nach Klassenreihenfolge die NRTI: 3TC, TDF, ABC und die PI: DRV, Atazanavir (ATV) und Ritonavir (r) als Boostersubstanz sowie für den Doppel-NRTI-Backbone (Backbones bilden die Basis einer Kombination, die mit weiteren Substanzen anderer Klassen gemeinsam verabreicht werden): TDF/FTC, ABC/3TC als Kombinationspräparate sowie einzeln kombiniertes 3TC/TDF an. Daraus ergeben sich folgende maximale Generika-zu-Original-Verhältnisse für die Mehrtabletten-Regime-Kombinationen: NNRTI/NRTI (1:1), INSTI/NRTI (1:1) und PI/NRTI (3:0), da die in den Leitlinien als zu bevorzugende Therapie empfohlenen NNRTI und INSTI noch nicht generisch verfügbar sind (Tab. 2).

**Table 2:** 

Für die Primärtherapie empfohlen:		DDD Ist		DDD Optional durch aufspalten	
		Original	Generisch	Original	Generisch
*Eintablettenregime*					
INSTI-basiert	BIC/TAF/FTC	1	–	–	–
	DTG/ABC/3TC	1	–	1	1
	DTG/3TC	1	–	1	1
	EVG/c/TAF/FTC	1	–	–	–
NNRTI-basiert	DOR/TDF/3TC	1	–	1	2
	RPV/TAF/FTC	1	–	–	–
	RPV/TDF/FTC	1	–	1	1
PI-basiert	DRV/c/TAF/FTC	1	–	–	–
*Mehrtablettenregime*					
INSTI-basiert	DTG + TAF/FTC	2	–	–	–
	DTG + TDF/FTC	1	1	–	–
	RAL + TAF/FTC	2	–	–	–
	RAL + (ABC/3TC oder TDF/FTC)	1	1	–	–
NNRTI-basiert	DOR + TAF/FTC	2	–	–	–
	DOR + (TDF/FTC oder ABC/3TC)	1	1	–	–
PI-basiert	DRV/r +TAF/FTC	1	2	–	–
	DRV/r + ABC/3TC	–	3	–	–

Durch Schrägstrich getrennte Substanzen sind in einem Präparat, durch Pluszeichen getrennte in verschiedenen Präparaten enthalten

*ABC* Abacavir, *BIC* Bictegravir, *c* Cobicistat, *DRV* Darunavir, *DDD* Defined Daily Dose, *DTG* Dolutegravir, *DOR* Doravirin, *EVG* Elvitegravir, *FTC* Emtricitabin, *INSTI* Integrase-Strang-Transfer-Inhibitoren, *3TC* Lamivudin, *NNRTI* Non-Nukleos(t)ide-Reverse-Transkriptase-Inhibitoren, *PI* Proteaseinhibitoren, *RAL* Raltegravir, *r* Ritonavir, *TAF* Tenofovir-Alafenamid, *TDF* Tenofovir-Disoproxil

Würden für den Doppel-NRTI-Backbone statt des generischen Kombinationsmedikaments die Einzelmedikamente genommen, kann pro Regime zulasten der Tablettenanzahl das Verhältnis um eine Generika-DDD gesteigert werden. Patentgeschützte Originale der Gruppe der Kombinationspräparate sind bis auf eine Ausnahme sämtlich Single-Tablet-Regimen (STR) und stellen jeweils eine nicht generische DDD pro Regime dar. Damit leisten STR einen negativen Beitrag im Sinne der Quote. Besonders ungünstig im Sinne der Generikaquote sind zusammengesetzte Regime mit dem Doppel-NRTI-Backbone, der Tenofovir-Alafenamid (TAF) enthält. Diese schlagen mit 2 nichtgenerischen DDD negativ zu Buche.

Es kann festgehalten werden, dass zusammengesetzte NNRTI- und INSTI-Regime mit Generikabackbone bezüglich der Generikaquote neutral sind, zusammengesetzte PI-Regime die Generikaquote durch Verdopplung oder Verdreifachung der DDD erhöhen bzw. dass diese ggf. 3 STR kompensieren. Daraus folgt, dass die in der KV Bayern (2020) festgesetzte Generikaquote von – für die meisten Arztgruppen grob zusammengefasst – etwa 43 % innerhalb der HIV-Therapieverordnungen nur mit einer ausreichend hohen Quote an zusammengesetzten PI-Regimen erreicht werden kann. Ausgleichend wirkt sich auch eine ausreichende Anzahl an HBV-Monoinfizierten und PrEP-Gebrauchern aus, die generisch behandelt werden.

**Figure 3:**
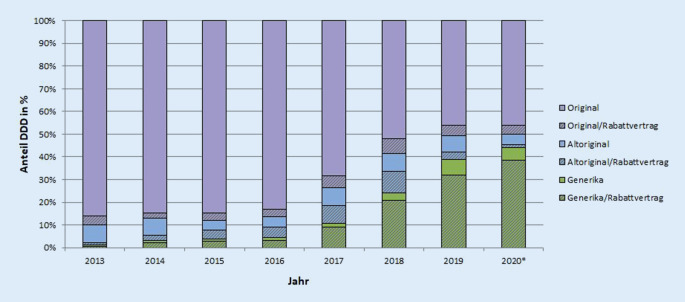

Im Datenkörper des DAPI sind Angaben zur Bindung der verordneten Medikamente an Rabattverträge verfügbar. Die Abb. 3 und 4 zeigen den bundesweiten Marktstatus und das Vorhandensein von Rabattverträgen.

**Figure 4:**
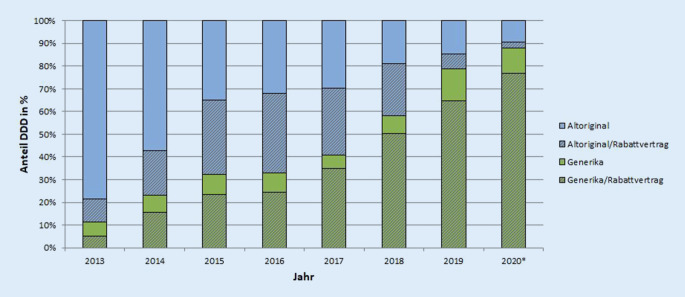

Mit dem Eintritt generischer Medikamente in den Markt, waren sowohl Verordnungen von rabattierten Generika als auch rabattierten Altoriginalen zu verzeichnen. Der Anteil an Verordnungen mit rabattierten Altoriginalen stieg über die Zeit an. Im Jahr 2016 überstieg das Segment der rabattierten Altoriginale sogar den gesamten Generikaanteil. Mit dem weiteren Anstieg der Generika im Verhältnis zu den Altoriginalen sank der Anteil von Altoriginalen mit Rabattbindung zuletzt in den Jahren 2019 und 2020 stark. Der Anteil von Altoriginalen ohne Rabattvertrag lag 2019 bei 7 % und 2020 bei 5 %. Die verordneten Generika waren über die gesamte Zeit fast vollständig Gegenstand von Rabattverträgen. In dem Kontext lassen sich auch Rückschlüsse auf Markteintrittsstrategien pharmazeutischer Unternehmen ziehen: Bei STR und patentgeschützten Medikamenten sind kaum Rabatte erkennbar. Anschließend, also ab generischer Verfügbarkeit, werden in größerem Umfang Rabatte auf das Altoriginal wie auch auf das Generikum gegeben. Im Kontext der jüngsten Einführung einer Festbetragsgruppe (ab Oktober 2020) kam es ebenfalls zum Abschluss von Rabattverträgen.

## Diskussion

Die verordnungssteuernden Maßnahmen in der GKV-Arzneimittelversorgung zeigen auch in der HIV-Schwerpunktversorgung Wirkung. Die in der Vergangenheit gängigen Steuerungsinstrumente, also statistische Auffälligkeitsprüfungen (zuvorderst Richtgrößen mit HIV als Praxisbesonderheit), werden dabei ergänzt oder gar ersetzt durch Quotenlösungen. Eine solche Steuerung setzt nicht beim Faktor Menge, sondern beim Faktor Struktur an. Die konkrete Ausgestaltung kann sehr unterschiedlich sein, dies zeigen die „Pionier“-Generikaquoten der KV Bayern und KV Berlin.

Eine Analyse auf Grundlage von GKV-Apothekenabrechnungsdaten verdeutlicht Potenziale, aber auch die Grenzen eines solchen Instrumentes zur Verordnungssteuerung. Vor allem 4 Sachverhalte zeigen sich:

1. Das Quotenpotenzial für Generika im patentfreien Bereich in der Versorgung von Menschen mit HIV wird bereits weitgehend ausgeschöpft. Dieser Umstand ist vor allem darauf zurückzuführen, dass die HIV-Verordner den Austausch mittels Verzicht auf Aut-idem-Kreuze unterstützen. Dass der Markteintritt von generischen HIV-Therapeutika im Verordnungsverhalten berücksichtigt wird, zeigen auch Umfragen [1], was im Ergebnis dazu führt, dass Rabattvertragsstrategien von Krankenkassen und Herstellern wirken können. Dadurch wird sowohl einerseits das Altoriginal zeitweise im Markt gehalten als anderseits die Abgabe des Generikums gesichert. Die Nichtverwendung von Aut-idem-Kreuzen durch die Verordner wirkt hier unterstützend. Grundsätzlich ist es daher sinnvoll, bei der Berechnung der Quoten Rabattverträge einzubeziehen.
2. Solange eine Generikaquote grundsätzlich anstrebt das generikafähige Potenzial auszureizen, d. h. den Anteil der nicht rabattierten Altoriginale zu substituieren, könnte eine (hypothetische) bundesweite Generikaquote bezogen auf den Gesamtmarkt HIV, PrEP und HBV um knapp 5 % (von aktuell ca. 44 %) gesteigert werden. Ob ein solcher Schritt mit Blick auf den geringen Preisunterschied zu den nichtrabattierten Altoriginalen wirtschaftlich sinnvoll ist, sei dahingestellt. Die bayerische Wirkstoffvereinbarung mit zumeist 49 % Quotenvorgabe dürfte damit bereits an Grenzen stoßen.
3. Ist man hingegen bereit, auch an die Wahl der verordneten Medikamente selbst heranzugehen, ergeben sich folgende Optionen: Die Generikaquote könnte gesteigert werden durch die „Aufspaltung“ von STR in einen generischen Backbone und einen originalen aktiven Teil, ferner durch die Auswahl nicht empfohlener generischer Klassenvertreter oder durch die Wahl einer Klasse mit hohem generischen DDD-Anteil (PI). Eine Abspaltung des generischen Backbones treibt im Ergebnis die Quote der generischen DDD, ist aber insbesondere nach Etablierung der entsprechenden Festbetragsgruppe wirtschaftlich nicht sinnvoll. Auf den Einspareffekt bezogen gibt es bei einigen Kombinationen keine nennenswerte Kostenersparnis bzw. ist die neue Kombination sogar teurer. Zudem sind die STR, die seit 2011 in den Markt kamen, alle AMNOG-bewertet.
4. Die HIV-Generikaquote ist de facto eine erweiterte Quote für den Gesamtmarkt HIV und HBV – inkl. der PrEP –, d. h., die Berechnung der Generikaquoten spiegelt den entsprechenden Gesamtmarkt wider und sollte inhaltlich und begrifflich entsprechend berücksichtigt werden. Beispielsweise ist die höhere Quote im HIV-Segment in Berlin vermutlich auf den höheren PrEP-Umsatz zurückzuführen.

Durch eine Orientierung an den erreichten Quoten werden diese auf dem Istniveau fixiert und es geht immer nur noch höher herauf (Soll-Zustand). Geht man davon aus, dass die bisherigen Quoten in einer ersten Phase eher dem „Gewöhnen“ der Verordner an das neue Steuerungsinstrumentarium dienen und zunächst weitgehend den Ist-Zustand abbilden, stellt sich die Frage, wie ein mögliches Soll aussehen könnte. Wie gezeigt werden konnte, besteht im Bereich der (nichtrabattierten) Altoriginale noch „Luft“. Hier ist allerdings zu bedenken, dass genau dieses Segment mittlerweile in Festbetragsgruppen eingeordnet wurde.

## Fazit

Die Steuerungsinstrumente der Arzneimittelversorgung in der GKV setzen auf indikationsbezogene Quoten, vorrangig Leitsubstanzen und/oder Generika/Biosimilars. Diese gibt es mittlerweile auch im HIV-Bereich, etwa die seit 2020 in Bayern und Berlin eingeführten Generikaquoten für HIV-Therapeutika. Ziel des vorliegenden Beitrages ist es, auf Grundlage von GKV-Apothekenabrechnungsdaten das Potenzial sowie Grenzen von Generikaquotenlösungen in der HIV-Versorgung zu analysieren und Handlungsempfehlungen zu skizzieren.

Es zeigt sich, dass das Quotenpotenzial für Generika im patentfreien Bereich in der HIV-Versorgung bereits weitgehend ausgeschöpft wird. Dieser Umstand ist vor allem darauf zurückzuführen, dass die HIV-Verordner den Austausch durch Verzicht auf Aut-idem-Kreuze auf dem Kassenrezept unterstützen. Das steuerungspolitische Optimum ist also fast erreicht. Dies ist ein Indiz dafür, dass ein Instrumentarium bestehend aus Rahmenvertrag zur Arzneimittelversorgung sowie einer leitliniengerechten Wirkstoffverordnung durch den Arzt – in Verbindung mit dem AMNOG und den Festbeträgen – steuerungspolitisch gut geeignet ist. Die Leitlinienkonformität und das (existierende) Eintablettenregime müssen aber beibehalten werden, damit die gute Versorgungsqualität gewährleistet bleibt. Die aktuellen Deutsch-Österreichischen Leitlinien streben als oberste Prinzipien virologische und immunologische Wirksamkeit sowie die Vermeidung von Resistenzentwicklung an [8]. Diese Ziele werden durch die Auswahl der ART anhand der Kriterien Anwendungsfreundlichkeit, geringe Einnahmefrequenz, niedrige Tablettenzahl, wenig pharmakokinetische Interaktionen und diätetische Restriktionen positiv beeinflusst.

Ein Austausch zum Erreichen einer höheren Generikaquote führt in jedem Fall zu einer Erhöhung der Tablettenanzahl und widerspricht damit dem Grundsatz der Anwendungsfreundlichkeit, ggf. führt er sogar zur Wahl einer suboptimalen Therapie. Dergestalt wäre eine Steuerung über Generikaquoten ein zu teuer erkaufter Erfolg. Im Tausch für mögliche wirtschaftliche Vorteile drohen dann Qualitätseinbußen in der Versorgung. Des Weiteren muss Raum für die Verordnung innovativer patentgeschützter Präparate bestehen, die das AMNOG-Verfahren durchlaufen haben (und damit aus Verordnersicht bei entsprechender Bewertung als wirtschaftlich gelten sollten [18]), was auch auf eine Absenkung von Quoten hinauslaufen kann. Hierbei sollte eine Quotenregelung therapeutischen Neuerungen und dynamischen Entwicklungen am Markt, nicht zuletzt bei HBV und PrEP (!) sowie einer individuellen Therapiewahl nicht im Weg stehen.

## Footnotes

4 Das Manuskript wurde im November 2020 eingereicht und konnte die in 2021 für die KV-Region Hamburg eingeführte Generikaquote daher nicht berücksichtigen.
